# Plant Gall Diversity in Burned Semi-natural Grasslands in Japan

**DOI:** 10.1093/jisesa/iead005

**Published:** 2023-02-02

**Authors:** Asuka Koyama, Tatsuya Ide

**Affiliations:** Center for Biodiversity and Climate Change, Forestry and Forest Products Research Institute, 1 Matsunosato, Tsukuba, Ibaraki 305-8687, Japan; Department of Zoology, National Museum of Nature and Science, 4–1–1 Amakubo, Tsukuba, Ibaraki 305–0005, Japan

**Keywords:** burning, cynipid gall wasp, insect-gall diversity, oak

## Abstract

We surveyed woody plants, including oaks and chestnuts (*Quercus* L. and *Castanea* Mill.), and recorded the inhabiting galls induced by oak gall wasps (Hymenoptera: Cynipidae: Cynipini) at seven Japanese semi-natural grassland sites maintained by traditional regular burning with two of the seven abandoned grassland areas. Woody plants were established in all burned and abandoned grasslands. Oak species were found at four of the seven sites. In total, 15 types of cynipid galls were recorded at all four sites where oak species were found. However, the occurrence of species was site-specific for host trees and cynipid galls. Although a few ecological studies of oak gall wasps inhabiting grassland environments, which have rapidly decreased in recent decades, have been conducted, this study suggests that semi-natural grasslands may be potential habitats for oak gall wasps and their host trees, and we provide a checklist of oak gall wasps with host oak records in semi-natural grasslands throughout Japan.

An estimated 21,000–211,000 (avg. 132,930) insect species can induce plant galls (Espírito-Santo and Fernandes 2007). A representative gall-inducing insect is cynipid gall wasps (Hymenoptera: Cynipidae), which are divided into woody rosid gallers, herb gallers, and inquilines, with the most diverse being woody rosid gallers, such as oak gall wasps (Ronquist et al. 2015). In Japan, secondary forests, which are often dominated by oak trees (Nakashizuka and Iida 1995), function as the primary habitat for oak gall wasps.

The plant vigor hypothesis (Price 1991) proposes that 1) gall-inducing insects preferentially oviposit on large, vigorously growing plant modules (such as shoots and leaves) and 2) this preference is selected for the increased fitness of these herbivores on vigorous modules compared with their fitness on less vigorous modules. In support of this hypothesis, several studies have shown that a plant resprouting after fire burning encourages higher colonization of galling insects (Vieira et al. 1996, de Souza Mendonça Jr. 2001, Andrade et al. 2019). In Japan, semi-natural grasslands traditionally managed by fire burning are often adjacent to deciduous oak forests, suggesting deciduous oak forests are associated with fire disturbance (Nakashizuka and Iida 1995, Ohwaki 2018). Such burned areas are potential habitats for cynipid gall wasps (Cronin et al. 2020). Those remind us that semi-natural grasslands managed by spring burning, which used to be the most widely distributed grassland type in Japan (Ushimaru et al. 2018), may also be preferred habitats for oak gall wasps but little is known about oaks and oak gall wasps inhabiting such environments.

In this study, to verify 1) the presence of host tree species and 2) the presence of cynipid galls in semi-natural grasslands, we provide a checklist of cynipid galls with woody-plant lists inhabiting semi-natural grasslands.

## Materials and Methods

### Study Area

**Fig. 1.**
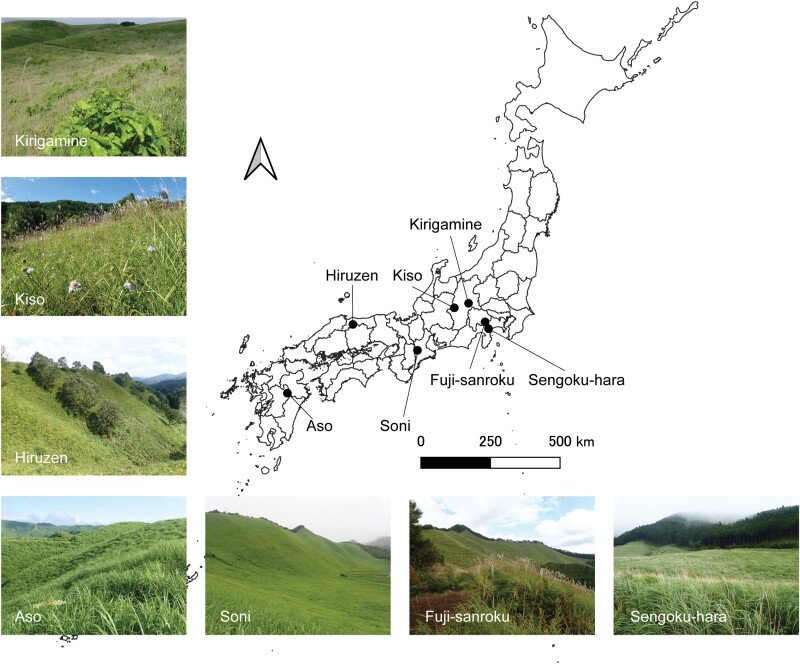
Study sites of seven semi-natural grasslands in Japan.

We selected seven mountainous and semi-natural grassland sites (Aso, Hiruzen, Soni, Kirigamine, Kiso, Sengoku-hara, and Fuji-sanroku) across Japan (Fig. 1). These sites have been traditionally managed through annual spring burning with or without seasonal mowing procedures and are now fragmented and surrounded by agricultural lands, plantations, and secondary forests due to land-use changes. The grasslands are in a range of climatic zones from warm temperate to cool temperate, with elevations ranging between 600 and 1,550 m (Supp File S1 [online only]). The dominance of grassland plant species, mainly *Miscanthus sinensis* Andersson, in these grasslands have been confirmed in previous studies (Koyama et al. 2018, Ohwaki et al. 2018, Koyama and Uchida 2022) or our preliminary study for Kirigamine (unpublished data). Grassland plant species are also dominant. The common host tree species of oak gall wasps (i.e., *Quercus acutissima* Carruth., *Q*. *crispula* Blume, *Q*. *dentata* Thunb., *Q*. *serrata* Murray, and *Castanea crenata* Siebold et Zucc.) can be climatically distributed across all studied regions.

### Woody-plant and Gall Surveys

We conducted woody-plant surveys to determine whether there were woody-plant species, including cynipid host tree species. During 2020–2021, two to six 10 m × 10 m plots were placed in each burned grassland site and subdivided into four 5 m × 5 m subplots. To investigate the effects of grassland abandonment, we set two similar plots at two sites (Hiruzen and Kiso) with surrounding abandoned grasslands. These were traditionally managed and have been abandoned for more than 15 yr. We recorded the species names of the emergent woody plants, including cynipid hosts. We also measured the maximum height of each species in each subplot and calculated the average maximum height at each site.

To examine whether and how many cynipid species are present in burned and abandoned grasslands, we conducted cynipid gall surveys in semi-natural grasslands, including adjacent forest edges. We took photographs of the galls on the host plants instead of collecting galls for species identification. Most oak gall wasps, which alternate generations, have sexual and asexual generation galls on different plant parts (Stone et al. 2002, Ide and Abe 2021). Therefore, we recorded each gall separately and organized the two types of galls by sexual and asexual generations of the same species into a single species based on Yukawa and Masuda (1996). We recorded not only young or matured galls but also old galls left on the trees after adult emergence. Although cynipid gall wasps contain herb gallers, none were found in this survey.

## Results

### Woody Plants and Inhabiting Galls of Semi-natural Grasslands

Woody plants appeared in all semi-natural grassland sites (Supp File S2 [online only]). In the burned plots, we counted 2–10 species per plot, and their height was less than 150 cm. *Lespedeza bicolor* Turcz. and *Rubus parvifolius* L. were predominant. In the abandoned plots, 4–16 species were present per plot, and their height was 220–300 cm. Host tree species appeared in four out of the seven semi-natural grasslands: all five host species in Hiruzen, three in Kiso, and one in Kirigamine and Fuji-sanroku. *Castanea crenata* was observed in three grasslands.

**Fig. 2.**
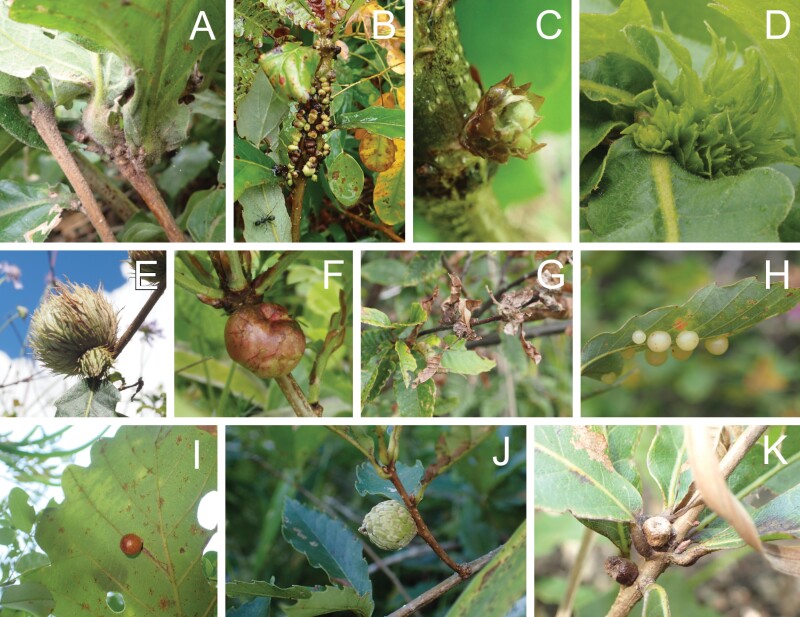
Cynipid galls found in semi-natural grasslands in Japan. (A) Sexual generation gall of *Andricus hakonensis* (23 September 2021, Hiruzen). (B) Asexual generation gall of *A*. *hakonensis* (25 September 2020, Hiruzen). (C) Sexual generation gall of *A*. *kashiwaphilus* (19 June 2020, Kirigamine). (D) Asexual generation gall of *A*. *kashiwaphilus* (20 June 2020, Kirigamine). (E) Asexual generation gall of *A*. *mukaigawae* (5 September 2020, Kiso). (F) Sexual generation gall of *Biorhiza nawai* (20 June 2020, Kirigimine). (G) Gall of *Dryocosmus kuriphilus* (16 September 2021, Fuji-sanroku). (H) Asexual generation gall of Cynipidae sp. 1 (16 September 2021, Fuji-sanroku). (I) Asexual generation gall of Cynipidae sp. 2 (22 September 2021, Hiruzen). (J) Asexual generation gall of Cynipidae sp. 3 (22 September 2021, Hiruzen). (K) Asexual generation gall of Cynipidae sp. 4 (23 September 2021, Hiruzen).

In total, 15 types of galls were recorded from five host tree species inhabiting four semi-natural grasslands, although the gall inducers of five of them were induced by unidentified cynipid species (Fig. 2). In Hiruzen, 10 types of galls were observed from four host tree species, seven from *Q. dentata* in Kirigamine, and three from *Q. serrata* in Kiso. Eight out of the 15 types of galls were recorded from one grassland.

### A Checklist of Cynipid Galls Recorded in the Semi-natural Grasslands in Japan

General characteristics of various galls are given in Supp File S3 (online only).

1) *Andricus hakonensis* (Ashmead)

Sexual generation gall (Japanese name: Nara-ha-guki-kobu-fushi; Fig. 2A): Kirigamine, 20. VI. 2020, *Q. dentata*; Hiruzen (burned and abandoned grasslands), 25. IX. 2020, 22–24. IX. 2021, *Q. dentata*.

Asexual generation gall (Japanese name: Nara-eda-mure-tama-fushi; Fig. 2B): Kirigamine, 20. VI. 2020, *Q. dentata*; Hiruzen (burned and abandoned grasslands), 25. IX. 2020, 22–24. IX. 2021, *Q. serrata*; Fuji-sanroku, 16. IX. 2021, *Q. serrata*.

2) *Andricus kashiwaphilus* Abe

Sexual generation gall (Japanese name: Kashiwa-waka-me-ko-mure-tama-fushi; Fig. 2C): Kirigamine, 19. VI. 2020, *Q*. *dentata*.

Asexual generation gall (Japanese name: Kashiwa-me-nise-hana-fushi; Fig. 2D): Kirigamine, 19–20. VI. 2020, *Q. dentata*.

3) *Andricus mukaigawae* (Mukaigawa)

Asexual generation gall (Japanese name: Nara-me-iga-fushi; Fig. 2E): Kiso (burned grassland), 5. IX. 2020, *Q. serrata*; Fuji-sanroku, 16. IX. 2021, *Q. serrata*.

4) *Andricus pseudocurvator* Tang & Melika

Sexual generation gall (Japanese name: Nara-ha-taiko-tama-fushi; not illustrated): Hiruzen (abandoned grassland), 22. IX. 2021, *Q. serrata*.

5) *Aphelonyx glanduliferae* (Mukaigawa)

Asexual generation gall (Japanese name: Nara-ha-ura-maru-tama-fushi; not illustrated): Hiruzen (abandoned grassland), 22. IX. 2021, *Q. serrata*.

6) *Biorhiza nawai* (Ashmead)

Sexual generation gall (Japanese name: Nara-me-ringo-fushi; Fig. 2F): Kirigamine, 20. VI. 2020, *Q. dentata*; Fuji-sanroku, 16. IX. 2021, *Q. serrata*.

7) *Cerroneuroterus japonicus* (Ashmead)

Asexual generation gall (Japanese name: Kunugi-ha-ke-tama-fushi; not illustrated): Hiruzen (burned grassland), 25. IX. 2020, *Q. acutissima*.

8) *Dryocosmus kuriphilus* Yasumatsu

Gall (Japanese name: Kuri-me-kobu-zui-fushi; Fig. 2G): Fuji-sanroku, 16. IX. 2021, *C. crenata*; Hiruzen (abandoned grassland), 22–24. IX. 2021, *C. crenata*.

9) Cynipidae sp. 1

Asexual generation gall (Japanese name: Nara-ha-hirata-maru-tama-fushi; Fig. 2H): *Q. serrata*; Hiruzen (burned and abandoned grasslands), 25. IX. 2020, 23–24. IX. 2021, *Q. serrata*; Fuji-sanroku, 16. IX. 2021, *Q. serrata*, *Q*. *crispula*.

10) Cynipidae sp. 2

Asexual generation gall (Japanese name: Kashiwa-ha-maru-oo-tama-fushi; Fig. 2I): Hiruzen (burned and abandoned grasslands), 25. IX. 2020, 22–23. IX. 2021, *Q. dentata*.

11) Cynipidae sp. 3

Asexual generation gall (Japanese name: Nara-me-uroko-tama-fushi; Fig. 2J): Kirigamine, 19. IX. 2020, *Q. dentata*; Hiruzen (burned and abandoned grasslands), 25. IX. 2020, 22–24. IX. 2021, *Q. serrata*; Fuji-sanroku, 16. IX. 2021, *Q. serrata*, *Q. crispula*.

12) Cynipidae sp. 4

Asexual generation gall (Japanese name: Nara-waka-me-hana-tsubo-tama-fushi; Fig. 2K): Hiruzen (burned grasslands), 23. IX. 2021. *Q. serrata*.

13) Cynipidae sp. 5

Gall (Japanese name: Nara-ha-ura-shiro-tama-fushi; not illustrated): Kirigamine, 19–20. VI. 2020, *Q. dentata*.

## Discussion

Woody-plant species sparsely inhabiting grasslands often increase heterogeneity and biodiversity (Milberg et al. 2020). For example, shrubs increase the abundance and reproduction of herbaceous plants due to grazing avoidance (Pihlgren and Lennartsson 2008), providing a habitat for insects and birds. Woody plants can also grow in traditionally and regularly burned semi-natural grasslands by shoot re-sprouting (Söderström et al. 2001), although the effect of woody plant species inhabiting semi-natural grasslands on biodiversity is often ignored. Plant galls are a good indicator for assessing such plant function and the diversity of other organisms (de Souza Mendonça 2007, Egan and Ott 2007, de Araújo et al. 2013).

We found that five cynipid host tree species, *Q. acutissima*, *Q. crispula*, *Q. dentata*, *Q. serrata*, and *C. crenata*, were established in four of the seven studied burned semi-natural grasslands in Japan. The presence of the five host species in burned grasslands was site-specific. Four out of the five species appeared in Hiruzen, three in Kiso, one in Kirigamine and Fuji-sanroku, whereas no host tree species were found in the other three grasslands. Although the determinants of host tree presence were not examined in this study, the lack of seed dispersal from surrounding forests, differences in historical and local management measures among grasslands, and impacts of herbivore grazing (such as deer) may affect the presence of host tree species.

We also found that trees inhabiting semi-natural grasslands act as hosts for various species of oak gall wasps. Their phenology would be synchronized with fire management timing and would take advantage of post-fire plant resprouting after fire burning as suggested by some galling insects (Vieira et al. 1996, de Souza Mendonça Jr. 2001, Andrade et al. 2019). In addition, the presence of various cynipid galls indicates the presence of various gall-inhabiting insects, such as gall inducers, inquilines, and parasitoids (Stone et al. 2002). Even though their potential habitats have rapidly decreased worldwide due to land-use transformation and management abandonment over the past few decades (Pykälä et al. 2005, Kirby et al. 2017, Koyama et al. 2017), research on the ecology, distribution, and conservation of the inhabiting tiny insects is poorly accumulated (Kim and Holt 2012). This study shed light on gall-inducing insects inhabiting traditional semi-natural grasslands and will promote further studies about them by providing a checklist of oak gall wasps with host oak records in semi-natural grasslands throughout Japan.

## Implication

Semi-natural grasslands are species-rich ecosystems traditionally managed using spring burning combined with mowing or grazing (Duelli and Obrist 2003). Over the past few decades, grassland management and restoration for biodiversity and landscape conservation have replaced traditional grassland use (Valkó et al. 2014, Koyama et al. 2018). Recent uniform management not intended for resource use often results in clearing woody plants from grasslands or understory vegetation in coppice forests. Grassland abandonment also appears to affect the diversity of oak gall wasps and the establishment of host tree species, although the data on abandoned grasslands are limited. Our results suggest that semi-natural grasslands maintained by traditional burning are important ecosystems for the biodiversity conservation of not only herbaceous grassland plants but also woody plants with inhabiting insects.

## Supplementary Material

iead005_suppl_Supplementary_Supp_S1

iead005_suppl_Supplementary_Supp_S2

iead005_suppl_Supplementary_Supp_S3
