# Supplementary material for: Plant Gall Diversity in Burned Semi-natural Grasslands in Japan
Source: J Insect Sci. 2023 Feb 2;23(1):5. doi: 10.1093/jisesa/iead005 (PMC9894004; doi:10.1093/jisesa/iead005)
Supplement: iead005_suppl_Supplementary_Supp_S3 [file iead005_suppl_supplementary_supp_s3.docx]

**Supp. File S3. General characteristics of galls found in the semi-natural grasslands in Japan**

**1) *Andricus hakonensis* (Ashmead)**

Sexual generation gall. Leaf veins and/or petioles are irregularly swollen and densely covered with minute setae. Immature galls appear yellowish green with reddish parts and mature galls appear pale green. One to 30 larval chambers are contained in a single gall. This gall is the same as that illustrated by Yukawa and Masuda (1996) in fig. C-070.

Asexual generation gall. Rounded-oblong galls are clustered on a twig. Each gall is 4–6 mm in diameter and 7–8 mm in height. The gall surface is smooth, pale green, or pinkish-red. A single larval chamber is present in the gall. This gall is the same as that illustrated by Yukawa and Masuda (1996) in fig. C-069.

**2) *Andricus kashiwaphilus* Abe**

Sexual generation gall. Small oval galls are clustered on a sprout. Each gall has a thin wall. The larval chamber does not separate from the outer wall. This gall is the same as that illustrated by Yukawa and Masuda (1996) in fig. C-064.

Asexual generation gall. The axillary bud is transformed into a flower-shaped gall. The coloration is almost the same as that of the healthy leaves. A single larval chamber is placed at the base of the leaflets. This gall is the same as that illustrated in Yukawa and Masuda (1996) in fig. C-066.

**3) *Andricus mukaigawae* (Mukaigawa)**

Asexual generation gall. The axillary bud is transformed into a bur-shaped gall. The coloration is almost the same as that of the healthy leaves. A single larval chamber is placed at the base of the stylets. This gall is the same as that illustrated in Yukawa and Masuda (1996) in fig. C-126.

**4) *Andricus pseudocurvator* Tang & Melika**

Sexual generation gall. A blister-like spherical gall with somewhat flat upper and lower surfaces is induced on the leaf blade. It sometimes appears pale green with a red circle. A single larval chamber is at the center of the gall and separated from the outer wall by air space. The larval chamber is connected to a leaf vein. This gall is the same as that illustrated by Yukawa and Masuda (1996) in fig. C-145.

**5) *Aphelonyx glanduliferae* (Mukaigawa)**

Asexual generation gall. A spherical gall is induced on the underside of the leaf vein. The surface appeared smooth and polished and white or pinkish-red. The outer wall is thin and not separated from the larval chamber. This gall is the same as that illustrated by Yukawa and Masuda (1996) in fig. C-143.

**6) *Biorhiza nawai* (Ashmead)**

Sexual generation gall. A bud swells into a globular gall, attaining a diameter of 50 mm. The gall contains 30–40 larval chambers. The outer wall is thick, spongy, and not separated from the larval chambers. The gall surface is pale green, yellowish white, or pinkish red. This gall is the same as that illustrated by Yukawa and Masuda (1996) in fig. C-134.

**7) *Cerroneuroterus japonicus* (Ashmead)**

Asexual generation gall. Multiple galls are induced on the underside of a single leaf, typically in a cluster. Each gall is globular, roundly depressed at the top, and covered with dense, minute white setae. They appeared yellowish-brown to reddish-brown. A single larval chamber is placed at the center of the gall. The outer wall is thick, spongy, and juicy when fresh. This gall is the same as that illustrated by Yukawa and Masuda (1996) in fig. C-088 and C-090.

**8) *Dryocosmus kuriphilus* Yasumatsu**

Gall. A bud or young sprout swells irregularly into the gall. The gall is accompanied by some original leaves. The outer gall is thick, packed with hard tissue, and woody when dry. Up to 10 larval chambers are contained in a single gall. This species is univoltine (Yukawa and Masuda 1996). This gall is the same as that illustrated by Yukawa and Masuda (1996) in fig. C-106.

**9) Cynipidae sp. 1**

Asexual generation gall. A hemispherical gall is induced on the leaf vein (primarily the leaf underside). The gall surface appeared smooth and whitish-yellow or pinkish-red. A single larval chamber is placed near the bottom of the gall. The outer wall is thick and juicy when fresh. This gall is the same as that illustrated by Yukawa and Masuda (1996) in fig. C-146.

**10) Cynipidae sp. 2**

Asexual generation gall. A globular gall is induced on a leaf vein. The surface is smooth with a pale green or pinkish-red color. A single larval chamber is placed at the center of the gall. The outer wall is thick and juicy when fresh. This gall is possibly the same as that illustrated by Yukawa and Masuda (1996) in fig. C-068 as kashiwa-ha-maru-tama-fushi.

**11) Cynipidae sp. 3**

Asexual generation gall. The bud is transformed into an oval gall. The gall surface is covered with scaly fragments. A single larval chamber is placed at the center of the gall. This gall is the same as that illustrated by Yukawa and Masuda (1996) in fig. C-186 as mizunara-me-uroko-tama-fushi.

**12) Cynipidae sp. 4**

Asexual generation gall. A bud transforms in to a single gall. The gall is cylindrical but narrowed near the top of the gall to form a pot-like shape. The gall surface is smooth and pinkish-red. A single larval chamber is placed near the bottom of the gall. This gall is the same as that illustrated by Yukawa and Masuda (1996) in fig. C-125.

**13) Cynipidae sp. 5**

Gall. A blister-like spherical gall is raised on the underside of the leaf. The gall surface is white or pale green. A single larval chamber is at the center of the gall. The outer wall is juicy when fresh. Mature galls drop from the leaf, leaving a small pore on the leaf. This species is considered a univoltine species (Yukawa and Masuda 1996). This gall is the same as that illustrated by Yukawa and Masuda (1996) in fig. C-141.
